# Effects of age-related changes in trunk and lower limb range of motion on gait

**DOI:** 10.1186/s12891-023-06301-4

**Published:** 2023-03-28

**Authors:** Meiling Zhai, Yongchao Huang, Shi Zhou, Yahong Jin, Jiayun Feng, Chaolei Pei, Li Wen, Li Wen’s

**Affiliations:** 1 School of Sports and Health, Nanjing Sport Institute, Nanjing, Jiangsu China; 2 Physical Activity, Sport and Exercise Research Theme, Faculty of Health, Southern Cross University, 2480 Lismore, NSW Australia; 3 School of Physical Education and Humanities, Nanjing Sport Institute, Nanjing, Jiangsu China; 4 Institute of Sports Training, Tianjin University of Sport, Tianjin, China; 5 Institute of Physical Education, Shanxi Normal University, Taiyuan, China

**Keywords:** Gait, Range of motion, Trunk, Older, Young

## Abstract

**Background:**

The ability to walk is crucial for maintaining independence and a high quality of life among older adults. Although gait characteristics have been extensively studied in older adults, most studies have investigated muscle activity in the joints of the trunk or the lower limbs without assessing their interactions. Thus, the causes of altered trunk and lower limb movement patterns in older adults remain to explore. Therefore, this study compared the joint kinematic parameters of both trunk and lower limbs between young and older adults to identify kinematic factors associated with changes in gait among older adults.

**Methods:**

In total, 64 older (32 males, aged 68.34 ± 7.38 years; 32 females, aged 67.16 ± 6.66 years) and 64 young (32 males, aged 19.44 ± 0.84 years; 32 females, aged 19.69 ± 0.86 years) healthy adults participated in this study. The range of motion (ROM) of the thorax, pelvis, and trunk in the horizontal plane and of the hip, knee, and ankle joints of the lower limbs in the sagittal plane were measured using a motion capture system with wearable sensors. Two-way analysis of variance assessed differences in ROM by group, sex, and spatio-temporal gait parameters; Pearson correlation analysis assessed the correlation of the trunk and lower limbs.

**Results:**

Step length, gait speed, and stride length were greater in young adults (p < 0.001) than in older adults, but older women displayed the fastest gait speed (p < 0.05). ROM values for the pelvis, thorax, trunk, knee joint, and ankle joint of young adults were greater (p < 0.05) than those in older adults. However, hip ROM in older adults was significantly greater than that in young adults (p < 0.05).

**Conclusion:**

With increasing age, ROM of the lower limbs, especially the ankle joint, decreased significantly, resulting in a significant decrease in gait speed. As ROM of the pelvis decreased, stride length decreased significantly in older adults, who compensate through thoracic rotation. Thus, older adults should enhance muscle strength and increase ROM to improve gait patterns.

## Background

Walking is an indispensable physical activity in the daily life of older adults, and the ability to walk is of considerable importance for maintaining independence among older adults [1]. However, during aging, the range of motion (ROM) of the hip, knee, and ankle joints decreases. A study by Joseph and colleagues showed that having an abnormal gait is a risk factor for falls, and that normal gait depends on the coordination of the articular muscle system [2]. With increasing age, the trunk becomes stiff, and the decrease in ROM of the thorax and pelvis may lead to decreasing muscle function and abnormal gait [3]. The decrease in ROM of the joints in the lower limbs, especially the knee joint, may disrupt the gait and affect the balance [4]. Thus, the effect of the articular muscle system degradation on gait in older adults may be assessed through an analysis of their trunk and lower limb ROM.

Chung et al. found that the movement of the trunk on a sagittal plane during walking balances the movement of the lower limbs [5]. In healthy adults, changing or limiting the movement of the trunk leads to compensatory foot movement [6]. Assessing how an individual walks is necessary to analyze the associated movements of the trunk and lower limbs in the gait. Due to differences in body structures and walking habits, males and females display different gait characteristics [7]. For example, the step length of a male is longer than that of a female, whereas the gait velocity of the female is higher [8]. Females have a more forward pelvis and hip flexion and a greater abduction angle of the knee joint in the gait than males [9]. Therefore, a more comprehensive understanding of sex differences in the gait of older adults may provide targeted exercise guidance.

Although gait characteristics have been extensively studied in older adults, most studies have investigated ROM in the joints of the trunk or the lower limbs without assessing their interactions [10, 11]. Thus, the causes of altered trunk and lower limb movement patterns in older adults remain to explore. Therefore, this study aimed to (1) test the effects of demographic factors, including sex and height, on ROM; (2) assess differences in ROM by age, sex, and basic gait parameters; and (3) assess the correlations of the trunk and lower limbs. We assume that there are significant differences in ROMs based on sex and age, leading to differences in spatio-temporal gait parameters.

## Methods

This study used a cross-sectional design comparing sex and age effects between groups to identify kinematics factors contributing to gait changes in older adults. The sample size required for adequate statistical power was estimated using G*Power, version 3.1.9.2, given the assumptions of α level 0.05, power 0.95, and effect size 0.4. The minimum sample size required was estimated as 84 for analyses using two-way analysis of variance. This study was approved by the Ethics Committee of Tianjin Institute of Physical Education (approval No. TJUS2021029). All participants provided written informed consent before the start of the study.

### Participants

In total, 64 older adults (32 males, 68.34 ± 7.38 years of age; 32 females, 67.16 ± 6.66 years of age) and 64 young adults (32 males, 19.44 ± 0.84 years of age; 32 females, 19.69 ± 0.86 years of age) volunteered to participate in this study. The inclusion criteria for participants were (1) good health and no history of injury in the past 3 months, (2) no musculoskeletal disease or loss of balance, and (3) the ability to walk independently without the help of equipment or another individual.

The participants were recruited from the local community through online recruitment. The young adults were university students and the older adults were residents of Tianjin City. Participants were screened for potential risk factors for musculoskeletal function using the Short Musculoskeletal Function Assessment [12] to ensure that all participants were free of severe spinal and lower limb musculoskeletal problems.

### Kinematic evaluation

**Fig. 1:**
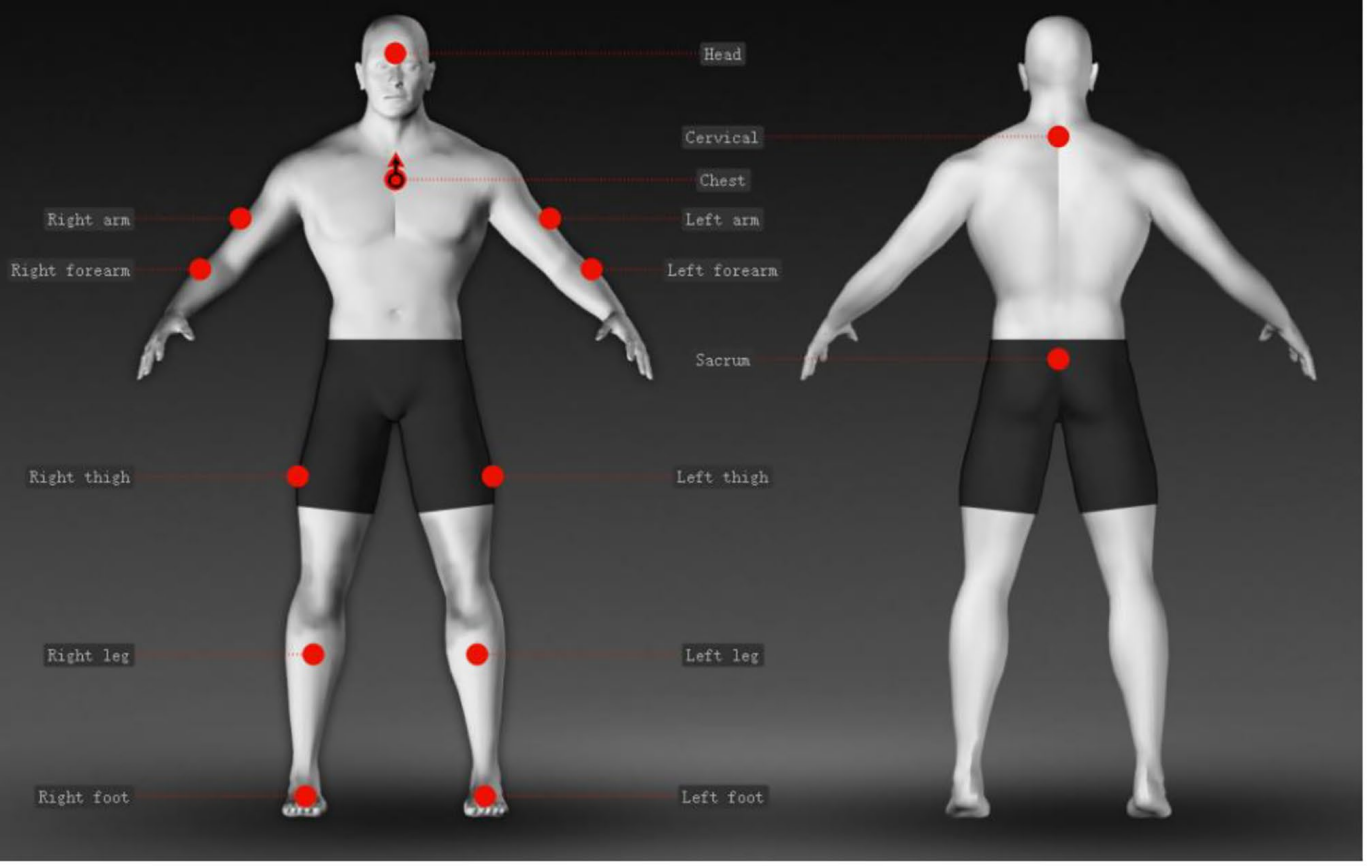
Sensor placemat map (from the STT motion capture system instruction manual)

**Fig. 2:**
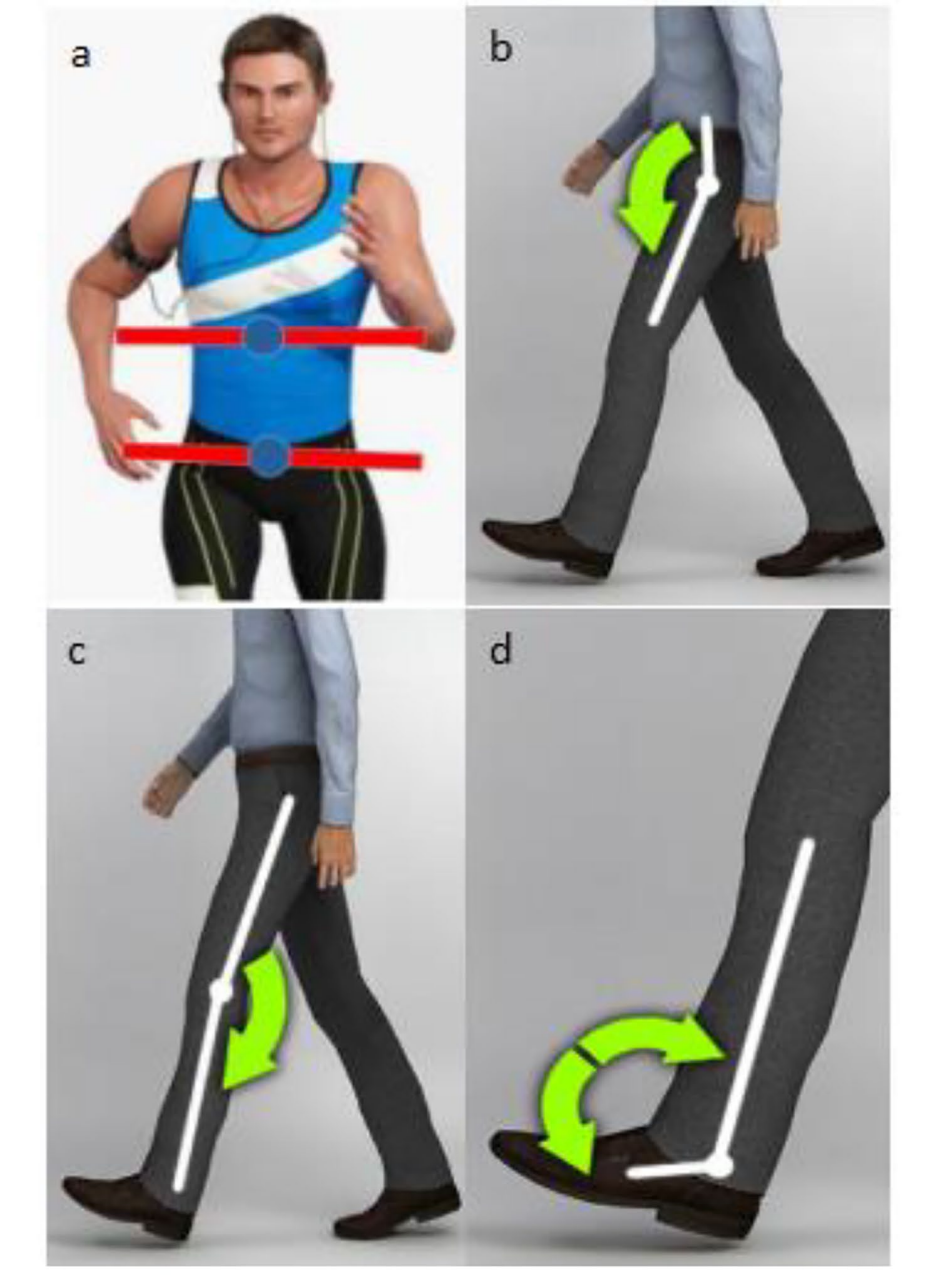
**a** Trunk rotation ROM. **b** Hip flexion/extension ROM. **c** Knee flexion/extension ROM. **d** Ankle plantarflexion/dorsiflexion ROM. (from the STT motion capture system instruction manual)

Joint kinematic parameters and spatio-temporal gait parameters were recorded using a motion capture system (STT Systems, iSEN 3.0; Spain). The use of a motion capture system with a wearable sensor has been shown to be reliable and valid in gait analysis [13]. As shown in Fig. 1, the inertial sensor was fixed on the seventh cervical spine, center of gravity of the human body (above the sacrum L4), forehead, first thoracic spinous process, manubrium sterni, bilateral anterior thigh, anterior calf, and foot surface to measure trunk rotation, hip flexion/extension, knee flexion/extension, and ankle plantarflexion/dorsiflexion (Fig. 2). The sampling frequency was 100.00 Hz. This test referenced as 10 m walking test (10MWT) for clearance. During the test, after hearing the word *start*, participants walked straight to the 10-m mark line at their chosen speed. In order to avoid interference and error, only data from the middle 6 m was collected. The experiments were repeated thrice and the mean values were calculated. The data were manually exported for processing and analysis after the walking exercise test was completed.

The software automatically calculated spatio-temporal gait parameters, including step length, stride length, cadence, and gait speed, by using foot surface markers to identify toe-off and heel strike events during the gait cycle. Step length was defined as the anteroposterior distance from one heel footprint to another heel footprint. Stride length was defined as the anteroposterior distance from two consecutive heels of the same foot. Cadence was calculated as the number of steps divided by time. Gait speed was calculated by dividing the walking distance by time. To avoid differences between individuals caused by height, normalized step length (i.e., step length divided by height) and stride length (i.e., stride length divided by height) were reported. ROM is the angle of the maximum absolute value in each phase of the gait cycle. Trunk ROM was defined as the absolute angular difference in degrees from the maximal (chest-pelvis relative rotation) to the minimal rotation within each cycle (heel-off to heel-off on the same leg).

### Division of the gait cycle

**Fig. 3:**
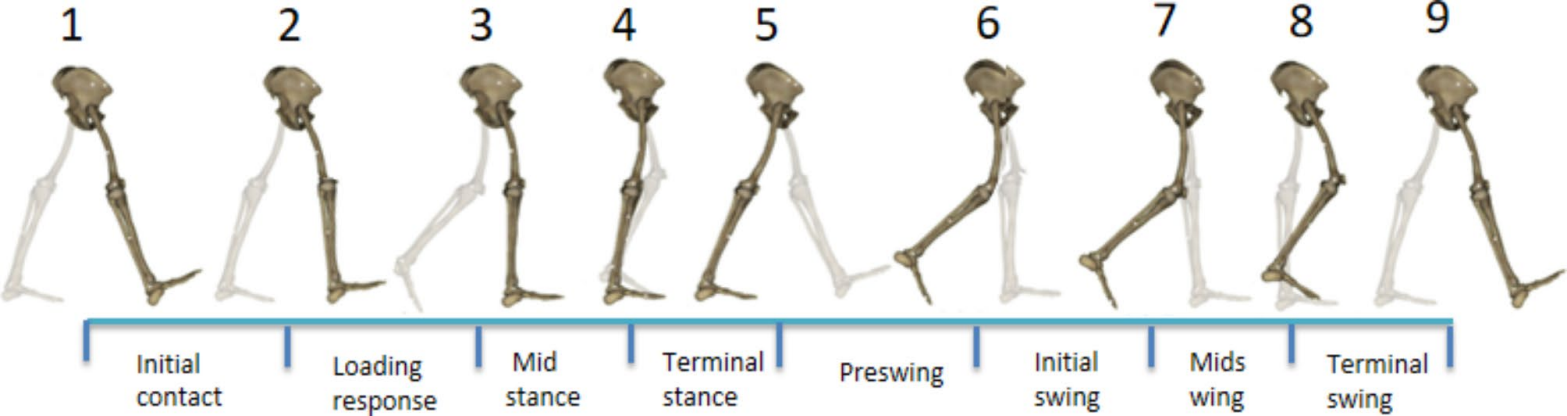
Division of gait cycle

This study used the classic 8-point method to divide the phases of the gate cycle [14]. As shown in Fig. 3, those 8 phases included the initial contact phase (0% of the gait cycle), loading response phase (0-10% of the gait cycle), mid-stance phase (10-30% of the gait cycle), terminal stance phase (30-50% of the gait cycle), pre-swing phase (50-60% of the gait cycle), initial swing phase (60-73% of the gait cycle), mid-swing phase (73-87% of the gait cycle), and terminal swing phase (87-100% of the gait cycle).

### Statistical analysis

The data were analyzed using IBM SPSS Statistics software (version 23.0, New York, USA). Each variable was described as the mean ± SD. The normality of the data distribution was assessed using the Shapiro-Wilk test, and the equality of the variances between groups was assessed using Levene’s homogeneity test. Multi-way ANOVA was used to test the effects of demographic factors, including sex and height, on ROM. Two-way ANOVA was used to assess differences in ROM by group, sex, and spatio-temporal gait parameters. Pearson correlation analysis was conducted to assess correlation between spatio-temporal gait parameters and ROM of the trunk and lower limb joints.

## Results

### Association of demographic characteristics with ROM

Demographic information for the 64 older adults and 64 young adults who volunteered to participate in this study are given in Table 1.

**Table 1:** Demographic characteristics of the participates

	Older adults (*n* = 64)			Young adults *(n* = 64)	
	Male (*n* = 32)	Female (*n* = 32)		Male (*n* = 32)	Female (*n* = 32)
Age (y) +	68.34 ± 7.38	67.16 ± 6.66		19.44 ± 0.84	19.69 ± 0.86
Height (m) *+	1.67 ± 4.37	1.58 ± 6.58		1.76 ± 3.84	1.63 ± 3.21
Weight (kg) *+	68.13 ± 5.67	56.41 ± 2.80		64.16 ± 5.59	50.50 ± 2.95
BMI(kg/m^2^) *+	24.52 ± 1.71	22.65 ± 2.01		20.69 ± 1.51	18.93 ± 1.50

* Represents a significant sex difference; + represents a significant difference between groups

As shown in Table 2, there was a statistically significant sex difference in hip ROM (F = 3.550, p < 0.05). With the exception of hip ROM, there were statistically significant differences between young and older adults (all p < 0.05). The interaction between age and sex was statistically significant for trunk ROM (F = 3.696, p < 0.05), knee ROM (F = 30.808, p < 0.05) and ankle ROM (F = 18.464, p < 0.05). By contrast, there was no statistically significant difference in ROM by height, weight, or body mass index (all p > 0.05).

**Table 2:** Multi-way ANOVA of participates’ basic information and ROM

	Trunk ROM	Chest ROM	Pelvis ROM	Hip ROM	Knee ROM	Ankle ROM
Height	0.121	2.415	0.355	0.221	2.961	1.203
Weight	0.002	1.782	0.425	0.116	2.006	1.444
BMI	0.007	1.550	0.546	0.039	2.673	0.817
Sex	1.300	2.146	0.360	3.550*	1.579	0.014
Group	9.126*	12.045*	107.364*	0.021	19.124*	50.856*
Sex*group	3.696*	0.549	1.419	0.424	30.808*	18.464*

* Represents a significant difference between the demographic characteristic and range of motion (ROM), *p* < 0.05

### Spatio-temporal gait parameters

As indicated in Table 3, there were statistically significant differences in step length (F = 6.876, p < 0.05), stride length (F = 12.859, p < 0.001), and gait speed (F = 4.620, p < 0.05) between age groups, with all three parameters showing higher values in young adults. There was a statistically significant interaction between sex and group for cadence (F = 4.751, p < 0.05). Further analysis showed that cadence in older adults was significantly higher than that in young adults (F = 13.646, p < 0.001) and significantly higher in females than in males (F = 7.087, p < 0.001).

**Table 3:** Comparison of gait parameters between sex and group

	Normalized Step length		Normalized Stride length		Cadence (steps/min)		Gait speed (m/s)	
	Male (n = 32)	Female (n = 32)	Male (n = 32)	Female (n = 32)	Male (n = 32)	Female (n = 32)	Male (n = 32)	Female (n = 32)
Older adult group (n = 64)	0.50 ± 0.15	0.52 ± 0.08	1.00 ± 0.28	1.04 ± 0.15	1.13 ± 3.87*	1.17 ± 5.98*+	1.39 ± 0.47	1.35 ± 0.43
Young adult group (n = 64)	0.55 ± 0.10	0.58 ± 0.12	1.09 ± 0.20	1.15 ± 0.26	1.12 ± 6.00	1.12 ± 6.29+	1.54 ± 0.37	1.53 ± 0.46

* Represents a significant sex difference; + represents a significant difference between groups only for the interaction

### Differences in ROM

As shown in Table 4, statistically significant age-related differences in ROM were observed for the pelvis (F = 232.340, p < 0.001) and thorax (F = 23.565, p < 0.001), with ROM in young adults significantly greater than that in older adults. We also observed a significant interaction between sex and group for trunk ROM (F = 5.772, p < 0.05). Further analysis showed that trunk ROM in young adult females was significantly greater than that in older adult females (F = 288.735, p < 0.001), and significantly greater than that in young adult males (F = 5.554, p < 0.05).

**Table 4:** Comparison of Trunk ROM between sex and group

	Pelvis ROM (degree)		Thoracic ROM (degree)		Trunk ROM (degree)	
	Male (*n* = 32)	Female (*n* = 32)	Male (*n* = 32)	Female (*n* = 32)	Male (*n* = 32)	Female (*n* = 32)
Older adult group (n = 64)	9.61 ± 2.97	9.39 ± 2.24	16.61 ± 5.69	16.45 ± 4.01	6.56 ± 1.32	6.05 ± 1.45+
Young adult group (n = 64)	17.34 ± 3.38	18.38 ± 3.65	20.25 ± 3.93	21.15 ± 5.52	6.86 ± 1.95*	8.02 ± 2.79*+

* Represents a significant sex difference; + represents aa significant difference between the groups only for the interaction

**Fig. 4:**
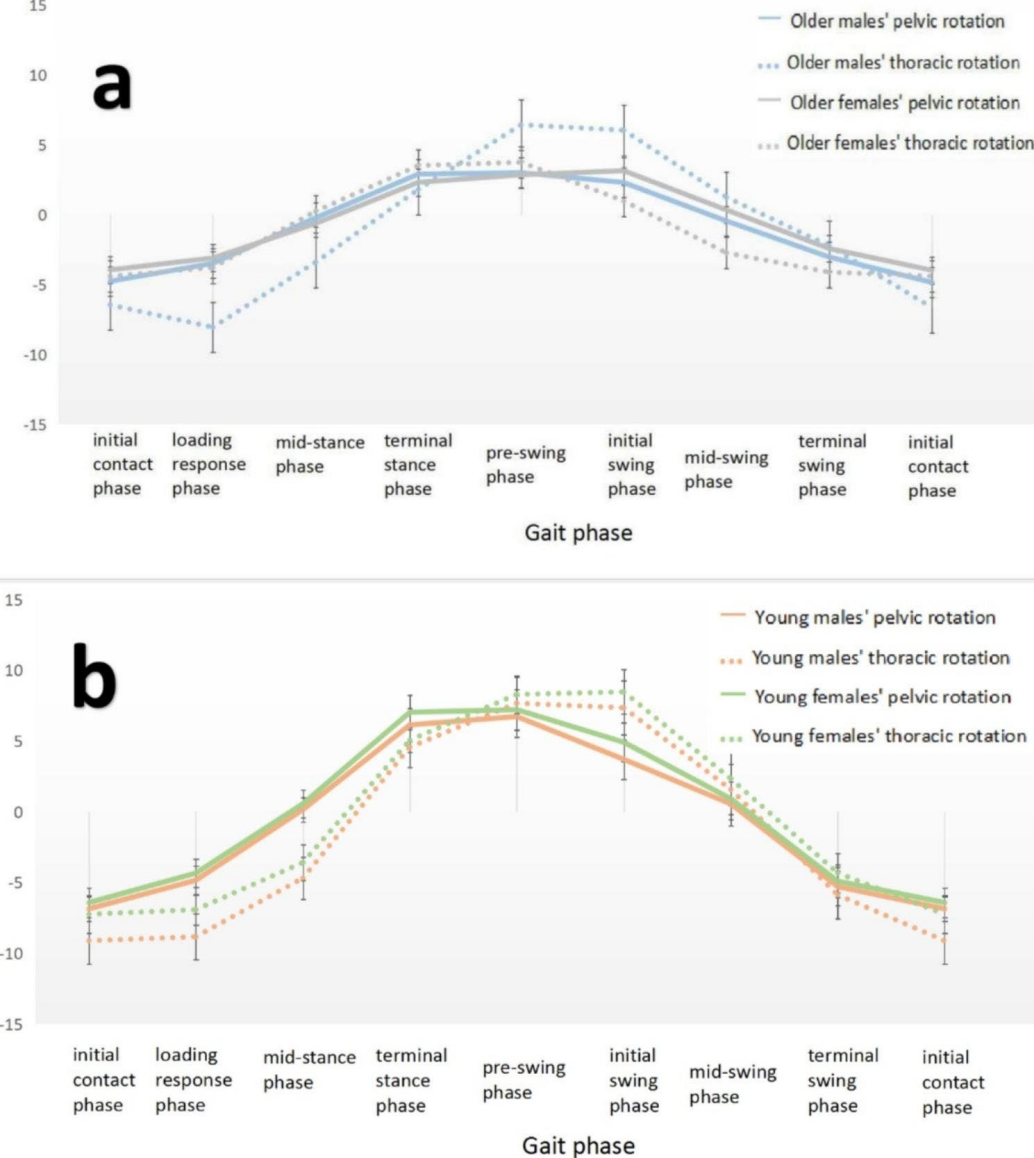
**a** Relative position curve in older male and older female thorax-pelvis. **b** Relative position curve in young male and young female thorax-pelvis. (Unit: degree)

Figure 4-a shows the anti-phase rotation of the thorax and pelvis from the initial contact phase to the loading response phase and from the terminal stance phase to the pre-swing phase in older male adults, with all other gait phases in phase. For older females, the anti-phase rotation of the thorax and pelvis occurred only during the pre-swing phase to the initial swing phase. Older males also showed greater variation in the thoracic rotation angle. As shown in Fig. 4-b, the anti-phase rotation of the thorax and pelvis in young males and females was from the terminal stance phase to the pre-swing phase, with good rotational symmetry of the trunk.

The results given in Table 5 indicated that hip ROM in older adults was significantly greater than that in young adults (F = 9.535, p < 0.05). In addition, hip ROM in males was significantly greater than that in females (F = 5.849, p < 0.05). There was a significant interaction between age and sex for knee ROM (F = 25.128, p < 0.001). Knee ROM in older males was significantly greater than that in females (F = 12.528, p < 0.05); knee ROM in young females was significantly greater than that in males (F = 5.554, p < 0.05); and knee ROM in young females was significantly greater than that in older females (F = 12.600, p < 0.05). Ankle ROM also showed a significant interaction between age and sex (F = 18.475, p < 0.001). Ankle ROM in young males was significantly greater than that in young females (F = 18.026, p < 0.05); ankle ROM in young adults was significantly greater than that in older adults (F = 68.412, p < 0.001).

**Table 5:** Comparison of lower limbs ROM between sex and group

	Hip ROM (degree)		Knee ROM (degree)		Ankle ROM (degree)	
	Male (*n* = 32)	Female (*n* = 32)	Male (*n* = 32)	Female (*n* = 32)	Male (*n* = 32)	Female (*n* = 32)
Older adult group (n = 64)	46.18 ± 5.93	43.95 ± 3.58	62.72 ± 4.05*	57.84 ± 4.96*+	29.60 ± 4.97+	32.83 ± 7.08+
Young adult group (n = 64)	44.48 ± 5.14	41.77 ± 2.79	62.74 ± 4.65*	67.63 ± 7.69*+	45.27 ± 9.83*+	37.78 ± 5.23*+

* Represents a significant sex difference; + represents a significant difference between groups only for the interaction

### Correlation between trunk and lower limb joints ROM in different gait phases

Table 6 shows that in the initial contact phase of the gait, trunk ROM was significantly and negatively correlated with ROM of the hip (r = − 0.379, p < 0.001) and knee (r = − 0.416, p < 0.001). During the loading response phase, trunk ROM was significantly and negatively correlated with ROM of the hip (r = − 0.200, p < 0.001), knee (r = − 0.401, p < 0.001) and ankle (r = − 0.160, p < 0.001). In the mid-stance phase, trunk ROM was significantly and negatively correlated with ROM of the hip (r = − 0.232, p < 0.001) and knee (r = − 0.354, p < 0.001).

**Table 6:** Correlation analysis between trunk and lower limb joints ROM in different gait phases

Trunk ROM in different gait phases	Hip ROM	Knee ROM	Ankle ROM
initial contact phase	-0.379**	-0.416**	-0.108
loading response phase	-0.200**	-0.401**	-0.160**
mid-stance phase	-0.232**	-0.354**	-0.005
terminal stance phase	0.251**	0.161**	-0.184**
pre-swing phase	0.609**	0.565**	-0.481**
initial swing phase	0.109**	0.136**	-0.156**
mid-swing phase	-0.053*	-0.011	-0.196**
swing phase	-0.074**	-0.189**	-0.263**

* *P* < 0.05; ** *P* < 0.001

For the terminal stance phase, trunk ROM was positively correlated with ROM of the hip (r = 0.251, P < 0.001) and knee (r = 0.161, P < 0.001), and negatively correlated with ankle ROM (r = − 0.184, P < 0.001). During pre-swing phase, trunk ROM was positively correlated with hip (r = 0.609, P < 0.001) and knee (r = 0.565, P < 0.001) ROM and negatively correlated with ankle ROM (r = − 0.481, P < 0.001). During the initial swing phase, trunk ROM was significantly and positively correlated with ROM of the hip (r = 0.109, p < 0.001) and knee (r = 0.136, p < 0.001), but significantly and negatively correlated with ankle ROM (r = − 0.156, p < 0.001). Therefore, in the terminal stance phase, the pre-swing phase, and the initial swing phase of gait, we found that trunk ROM, hip flexion angle, and knee flexion angle increased, while the ankle plantar flexion angle decreased.

During the mid-swing phase, trunk ROM was negatively correlated with ROM of the hip (r = − 0.153, p < 0.05) and ankle (r = − 0.196, p < 0.001). During the terminal swing phase, trunk ROM was significantly and negatively correlated with ROM of the hip (r = − 0.074, p < 0.001), knee (r = − 0.189, p < 0.001), and ankle (r = − 0.263, p < 0.001).

The results given in Table 7 indicate that there was a significant negative correlation between step length and thoracic ROM (r = − 0.256, p < 0.001). Stride length was positively correlated with pelvis ROM (r = 0.162, p < 0.05) and negatively correlated with thoracic ROM (r = − 0.243, p < 0.001). cadence was positively correlated with thoracic ROM (r = 0.182, p < 0.05) but negatively correlated with ROM of the pelvis (r = − 0.160, p < 0.05, trunk (r = − 0.169, p < 0.05) and knee (r = − 0.248, p < 0.001). Gait speed was positively correlated with ROM of the pelvis (r = 0.192, p < 0.05), hip (r = 0.192, p < 0.05), and ankle (r = 0.168, p < 0.05).

**Table 7:** Pearson correlation analysis in ROM and spatio-temporal gait parameters

ROM	Step length (m)	Stride length (m)	Cadence (steps/min)	Gait speed (m/s)
Thoracic ROM	-0.256**	-0.243**	0.182*	-0.063
Pelvis ROM	0.143	0.162*	-0.160*	0.192*
Trunk ROM	0.027	0.104	-0.169*	0.140
Hip ROM	0.045	0.101	-0.043	0.192*
Knee ROM	0.033	0.022	-0.248**	0.038
Ankle ROM	0.063	0.084	-0.070	0.168*

* *P* < 0.05; ** *P* < 0.001

## Discussion

### Comparison of gait parameters

The findings in this study indicated that older adults (mean age 68.34 ± 7.38 years) used more conservative and prudent gait strategies than young adults (mean age 19.44 ± 0.84 years) to maintain dynamic balance and prevent falls by reducing their gait speed and having a shorter stride length [15]. We found that older adults had a smaller step length, stride length, although older female displayed the fastest gait speed. The movement patterns of the trunk in the gait may explain the spatiotemporal changes we observed in the gait of older adults, such as their reduction in step length [16] and gait speed [17].

### Relationship between trunk ROM and basic gait parameters

Our results showed a positive, but not statistically significant, correlation between trunk ROM and step length or gait speed. It may be that the self-selected speeds in the present study were insufficient to increase trunk ROM; thus, the difference was not apparent. Future research should include additional challenging gait speeds to assess the effect of gait speed on trunk ROM. Pelvic ROM contributed little to gait lengthstep length [18], but it was significantly and positively correlated with stride length. By contrast, thorax ROM was significantly and negatively correlated with stride length. Although older adults showed a lower pelvic ROM during the gait cycle, they compensated with increased chest rotation.

Research has shown that as gait speed increases, trunk rotation changes from in-phase rotation to anti-phase rotation [19]. However, Bruijn et al. found that the thorax is more or less counter-rotated relative to the pelvis at all speeds [20]. We found that the thorax and pelvis rotated in opposite directions, and that there were statistically significant age-related and sex-related differences, although at comfortable gait speeds. Compared with that for older adults, gait speed was greater for young adults.

### Relationship between lower limb ROM and basic gait parameters

Regarding changes in the gait biomechanics of older adults, decreases in peak hip joint extension, ankle plantarflexion, and ankle muscle activation are related to aging [21]. The joints move primarily in the sagittal plane to push the body forward [22]. The decrease in ankle ROM in older adults may be correlated to a decrease in muscle strength and muscle activation of the related muscles, resulting in a lower pedal strength on the ground than that of young adults. Compared with young adults, older adults tend to draw their hips in a higher degree of extension, which is reflected in the larger extension range of the hip joint and the lower ankle ROM in the gait of older adults [23].

Segal et al. [24] found that when older adults walk at a speed similar to that of young people, they increase their gait speed by increasing the contribution from their hips. Therefore, a reduction in hip ROM is an important mechanism underlying the reduction in gait speed [25] and also a shorter stride length [26]. Our results suggested a positive correlation between hip ROM and step length, stride length, and gait speed, but only the correlation with gait speed was statistically significant. One study showed that knee arthrodesis appeared to reduce knee ROM, resulting in decreased gait speed, step length, and stride length [27], but the findings were not statistically significant. Doyo et al. [28] found that gait speed decreased with age, and that gait speed for females was higher than that for males. In the present study, knee ROM was found to be significantly and negatively correlated with gait speed. Older females had the fastest gait speed, which was slightly higher in older adults than in young adults. Perhaps sample sizes, participant characteristics, and choice of measurement instruments affected the results assessing age-related gait speed changes. More importantly, individuals with reduced ankle ROM showed shorter step length [29] and slower gait speed [30]. Lower limb strength training can result in a more upright gait, that is, increased hip and knee extension during the support phase of the gait [31].

Our results indicated that the coordinated between the trunk and lower limbs was different throughout the gait cycle phases. From the terminal stance phase to the initial swing phase of the gait, the ROM of the hip and knee joints increased significantly as the ROM of the trunk increased. At this time, the lower limbs mainly through the hip, knee swing and coordinate with the trunk. In the initial contact stage of gait, the foot is centered on the ankle joint, and the part above the ankle joint is the radius to carry out “pendulum” movement and coordinate with the trunk [32]. Furthermore, Olney et al. [33] found that active extension and flexion of the hip joint are important driving forces for maintaining trunk balance during walking in normal adults.

### Limitations and future research directions

Our study has limitations. First, our findings in a small sample of asymptomatic young and older adults may suggest early indications of age-dependent changes in joint mobility. Future research should include larger sample sizes across multiple age groups to determine when and which changes in joint mobility occur over time. Second, the effect of gate speed variation on ROM was not explored. In future research, gait speed should be included as an independent variable to explore the effect of its variation on gait kinematics. Third, we collected only kinematic parameters for the trunk and lower limbs; other kinetic parameters and muscle activity parameters with additional relevant variables should be included in future studies.

## Conclusion

ROM of the hip, knee, and especially the ankle decreased in older adults compared with young adults, resulting in a significant decrease in gait speed. Pelvic rotation had a significant effect on step length, and stride length decreased significantly in older adults with decreased mobility of the pelvis. To improve ROM of the pelvis, older adults compensated through thorax rotation. Therefore, older adults should enhance muscle strength and increase their ROM to improve their gait patterns.

## Data Availability

The data in this study are available from the corresponding author upon request, if legally and ethically possible. Each author warrants that this work is original. Neither this work nor a similar work by the authors has been published elsewhere in any language nor shall be submitted for publication elsewhere while under consideration by *BMC Musculoskeletal Disorders*.

## Abbreviations

ROM: range of motion
